# Rapid discrimination of *Panax ginseng* powder adulterated with various root plants by FT-IR spectroscopy coupled with multivariate analysis

**DOI:** 10.1007/s10068-023-01423-w

**Published:** 2023-11-17

**Authors:** Ji-Young Choi, Minhyun Kim, Sanghyeok Park, Jeong-Seok Cho, Jeong Ho Lim, Kwang-Deog Moon

**Affiliations:** 1 Food Safety and Distribution Research Group, Korea Food Research Institute, 245 Nongsaengmyeong-Ro, Wanju-gun, 55365 South Korea; 2 School of Food Science & Biotechnology, Kyungpook National University, 80 Daehak-Ro, Daegu, 41566 South Korea

**Keywords:** Ginseng products, Fourier transform infrared spectroscopy, Ginsenoside, Partial least squares regression, Principal component analysis

## Abstract

*Panax ginseng* powder adulterated with other root plants (arrowroot, bellflower, and lance asiabell) was discriminated using Fourier transform infrared (FT-IR) spectroscopy, combined with multivariate analysis. Principal component analysis visually diagnosed the adulteration by showing two distinct clusters based on presence of adulteration. Wavenumber regions (1000 cm^−1^ and 3300 cm^−1^) selected from the loading plot associated with the vibration of OH and CH bond in ginsenoside and aromatic compounds. A quantitative model for the content of ginsenosides and specific aromatic compounds as indicators of pure ginseng powder, was developed based on partial least square regression analysis. The performance of the prediction model preprocessed with the Savizky–Golay 1st derivative was improved to R^2^ of 0.9650, 0.9635, and 0.9591 for Rb1, Rc, and β-Panasinsene, respectively. Therefore, FT-IR technology makes it possible to rapidly authenticate pure ginseng product based on the ginsenoside contents and aroma compound.

## Introduction

*Panax ginseng* C.A. Meyer has been a popular herbal medicine throughout East Asia for thousands of years (Li et al., 2015) due to its various components, including ginsenosides, volatile oil, amino acids and peptides, polysaccharides, nitrogen compounds, and polyacetylenes (Yeo et al., 2017). In South Korea, ginseng and its related products are widely consumed as health supplements, general food, medicine, and the majority (more than 90%) is consumed as health foods (agricultural products, health supplements, and general food) including powder, tablet, beverage, and candy, (Baeg and So, 2013). As ginseng requires a long cultivation period of about 4 to 6 years to harvest, it is more expensive than other related crops (Chang et al., 2003). Hence, occurrence of adulteration by other cheaper products that are similar in properties (flavor, appearance, and components) is increasing and there is need for measures for quality assurance of ginseng.

The main indicator component of ginseng is triterpene glycoside, commonly called ginsenoside (Nam et al., 2013). Conventional analysis method of ginsenoside using high-performance liquid chromatography (HPLC) is time consuming, nonecofriendly, and requires trained researchers and complicated procedures. Additionally, the Korea Food and Drug Administration (2012) recommends use of genetic analysis to discriminate ginseng, lance asiabell, bellflower, and hemp from each other. This method also involves sample pretreatment, gene extraction, and many reagents. Engineers and food scientists around the world have developed efficient expertise and methodologies to investigate authenticity of ginseng products. Among the various analysis methods, spectroscopic (NIR, FT-IR, MIR) approaches attempt to detect the adulteration nondestructively (Jaiswal et al., 2018; Upadhyay et al., 2018). However, nondestructive, simple, and rapid analyses to identify quality fraud of ginseng-processed products remains a challenge. The use of spectroscopic techniques as a means to determine quality characteristics such as country of origin and cultivation age of ginseng has been widely investigated (Table 1). However, to date, adulteration identification of ginseng products using spectroscopic technique coupled with multivariate analysis remains a challenge.

**Table 1:** Summary of spectroscopic approaches used to identify the quality of ginseng products

Spectroscopic technology	Analysis method	Objective	References
FT-IR	2D-IR correlation spectra	Identification of American ginseng from different regions	Li et al. (2004)
FT-IR	2D-IR correlation spectra	Differentiation of Asian ginseng, American ginseng and Notoginseng	Lu et al. (2008)
FT-IR	2D-IR correlation spectra	Differentiation of the root of cultivated ginseng, mountain cultivated ginseng, and mountain wild ginseng	Liu et al. (2008)
IR	PCA	Authentication of Ginseng Species	Yap et al. (2009)
FT-IR	SIMCA, 2D-IR correlation spectra	Evaluation of cultivation types and the growth years (grade)	Zhang et al. (2010)
NIR	PLS	Estimation of concentration of ginsenosides in Asian ginseng	Inagaki et al. (2019)
FT-NIR	PLS, PCR	Determination the amount of ginsenosides in ginseng	Xu et al. (2014)
FT-IR	PLS	Discrimination and prediction of cultivation age and parts of *Panax* ginseng	Lee et al. (2017)
Near-infrared spectroscopy	PCA, PLS-DA, SIMCA, SPA-LDA	Identification of ginseng according to geographical origin	Chen et al. (2020)

Food fraud, the addition of an adulterant or a nondeclared substance, is attracting increasing attention as an emerging risk, because of the complex and global nature of food supply chains (Callo and Ruisánchez, 2018). A major concern about adulteration is that it may involve a health risk or an economic benefit. Thus, the major objective of this study was to identify adulteration of ginseng powder with other cheaper root plants by FT-IR spectroscopy combined with chemometric treatment. In this study, arrowroot, bellflower, and lance asiabell, which are root foods with similar characteristics to ginseng and cheaper than ginseng, were selected as adulteration materials. Subobjectives were as follows: to observe and compare the characteristics of FT-IR spectra information for ginseng powder and adulterated ginseng powder. Through principal component analysis, an influential key wavelength was obtained in the collected spectra data and the components of ginseng powder related to that region were analyzed. Finally, the optimal data preprocessing method was explored, and a partial least squares (PLS) regression model for predicting components of ginseng powder was used to assess the accuracy of component prediction for discrimination of adulterated ginseng powder.

## Materials and methods

### Sample preparation

Washed fresh ginseng grown for 5–6 years in Songnisan-myeon, Boeun, Chungbuk, South Korea, was purchased and ground into pure ginseng powder (RG) and dried. Lateral roots were separated from the main stem in fresh ginseng, and the main root was cut to a thickness of 5 mm and dried for 7 h in a hot air dryer (Kiturami drying machine, KED-132A, Kiturami, Seoul, Korea) at 50 °C. The resultant powder, with moisture content of about 9%, was sieved through an 850 μm mesh, and the fine powder was used as samples. Bellflower root (*Platycodon grandifloras*; Puleundeulpan, Seoul, Korea), arrowroot (*Pueraria hirsuta* Matsum, Puleundeulpan, Seoul, Korea), and lance asiabell (*Codonopsis lanceolata* Trautv., INCHA, Seoul, Korea), which were produced and dried in South Korea in 2021, were purchased for sample preparation. Dried bellflower, arrowroot, and lance asiabell were pulverized through a pulverizer (SNSG-1002SS, Hanil, Seoul, Korea) and those sieved through an 850 μm mesh were used as samples. Bellflower, arrowroot, and lance asiabell powders prepared in this way were named BF, AR, and LA, respectively. The moisture contents of the BF, AR, and LA powders were approximately 7%, 5%, and 7%, respectively. Ginseng powder-bellflower powder (GBF), ginseng powder-arrowroot powder (GAR), and ginseng powder-lance asiabell powder (GLA) were prepared by mixing pure ginseng powder and the other powder in a ratio of 7:3 for 7 min using a stomacher set to speed 4 (BagMixer 400, Interscience, Paris, France).

### Chemicals

Methanol and brutanol were obtained from Duksan Pure Chemicals Co. Ltd., Ansan, South Korea, for use in extracting saponin. Methanol for HPLC was obtained from Sigma-Aldrich, St Louis, MO, USA. Among the various ginsenoide types, the RG, Rb1 and Rc (Ace EMzyme Co., Anseong-si, Korea) were obtained as standards for ginsenoside HPLC analysis. Additionally, carbazole was obtained from Samchun Pure Chemicals Co., Seoul, Korea, for use in determining acidic polysaccharide content.

### Physicochemical analysis

#### Color

Color was measured to confirm the difference in the appearance of the samples. The powder was immersed in a petri dish with a diameter of 10 cm before its color was measured repeatedly for ten times per sample with a colorimeter (CR-400, Minolta, Tokyo, Japan). The colorimeter was calibrated to L^*^(lightness) = 97.79, a^*^(redness) =  − 0.38, and b^*^(yellowness) = 2.05.

#### Crude saponin and ginsenoside contents

Crude saponin content was measured by the method described by Park et al. (2009) with some modificactions. A total of 80 mL of 70% methanol was added to 4 g of the sample powder and extracted at a speed of 180 rpm for 4 h at 60 °C in a shaking incubator (JSSI-300C, JS research, Gongju, South Korea). The extract was filtered through Whatman no. 4 paper before methanol was evaporated using a rotary evaporator (RV 10 digital, IKA, Staufen, German). The dried material left was dissolved in 100 mL of water. After transferring it to a separatory funnel, 80 mL n-butanol was added to the separatory funnel, shaken, and left to stand. The n-butanol layer obtained by performing this process three times was collected and the solvent was evaporated on a rotary evaporator. The round bottom flask was dried at 55 °C ± 5 °C for 3–4 h before it was allowed to cool in a desiccator. The saponin content was calculated by Eq. (1).

1 $$\mathrm{Crude\,saponin\,contents }(\mathrm{mg}/\mathrm{g})=\frac{\mathrm{Weight\,of\,flask\,after\,drying }\left(\mathrm{mg}\right)-\mathrm{ Weight\,of\,flask }(\mathrm{mg})}{Sample (g)}$$

For the analysis of the content of Rg1, Rb1, Rc (called major ginsenoside) in saponin, 100 times the weight of the saponin separated above was dissolved in methanol for HPLC (Sigma-Aldrich) and used as a test solution. A volume of 10 μL of the sample was used for ginsenoside content analysis using HPLC (Model Prominence, Shimadzu, Kyoto, Japan). A concentration gradient of distilled water (solvent A) and acetonitrile (solvent B) based on solvent A was set for the mobile phase as follows: 90% (0 min), 60% (35 min), 60% (45 min), 90% (50 min), and 90% (60 min). C18 (150 × 4.6 mm, Waters Co., MA, USA) with a diameter of 5 µm was used for the column. The column temperature was set to 30 °C and the flow rate was set to 1.0 mL/min. Absorbance was measured at 203 nm with a DAD detector.

#### Acidic polysaccharides contents

As the acidic polysaccharide of Korean ginseng is mainly a polymer of galacturonic acid, which is similar to pectin in its molecular structure, it was measured using the carbazole–sulfuric acid method used for pectin quantification (Do et al., 1993). After adding 0.25 mL of carbazole and 3 mL of concentrated sulfuric acid to 0.5 mL of the ginseng solution, they were reacted for 5 min in a water bath at 80 °C. The mixture was left to cool at room temperature for 20 min before absorbance was measured at 525 nm with a UV–visible spectrophotometer. Galacturonic acid (Fluka Chemical Co.) was used as the standard material to prepare the standard curve.

#### Volatile flavor compounds

Volatile aroma compounds were measured using a 7890B gas chromatograph (GC) connected to a 5977B mass selective detector (Agilent Technologies, Inc., Santa Clara, CA, USA). The volatile aromatic components of ginseng powder and mixed powders were analyzed by GC–MS using the solid-phase microextraction method. Helium was used as the carrier gas, and electron ionization was the ion source. The flow rate of the carrier gas was maintained at 1 mL/min. The column was used to separate volatile aromatic compounds using DB-WAX (60 m × 0.25 mm id., 0.25-µm film thickness; J&W Scientific, Folsom, CA, USA). Afterward, 2.2 g of the sample was placed in a 20 mL vial and maintained at 70 °C for 20 min, followed by adsorption on fiber for 30 min. The oven temperature was maintained at 40 °C for 2 min before it was initially increased to 220 °C at a rate of 2 °C/min, and then to 240 °C at a rate of 20 °C/min, and maintained for 10 min. The total run time was 103 min. The temperature of the GC injector was set at 250 °C and the sample was injected at a split ratio of 1:20.

### Infrared spectra analysis

#### Spectra acquisition and data preprogressing

Fourier transform infrared (FT-IR; Nicolet™ iS5 FT-IR Spectrometer with iD7 attenuated total reflectance accessory FT-IR, Thermo Scientific, USA) analysis was performed to detected adulteration in dried ginseng powder. FT-IT is useful in quantitative analysis as it allows continuous monitoring of spectral baseline and aids in the simultaneous analysis of numerous parameters of the same sample (Bunaciu et al., 2009). FT-IR spectra were obtained in the range 4000 cm^−1^ to 550 cm^−1^. Spectra were recorded at a resolution of 4 cm^−1^ with 16 scans. Background calibration was performed using air blank references before measurement. Peaks caused by external influences were corrected through background collection and the spectrum of each sample was measured in 20 replicates. teh system was cleaned before and after each measurement using distilled water to wipe the ATR crystal. Spectral data were recorded using the OMNIC software (Thermo Fisher Scientific Inc., USA).

Spectra of various ginseng powders were preprocessed by smoothing, multiplicative scatter correction (MSC), standard normal variate (SNV), and Savitzky–Golay first derivative (SG-1) methods for scattering correction and noise removal. Data were preprocessed to improve the accuracy of component prediction models developed from PLS analysis.

#### Multivariate analysis

Multivariate analysis is mainly divided into unsupervised analysis, which finds hidden structures in data using no labeled data, and unsupervised analysis, which predicts outcomes by training labeled data. Principal component analysis (PCA), one of the supervised analysis methods, was performed to visualize overall clustering patterns of samples. Two-dimensional PCA score plots were created on the spectral data in the 3750–550 cm^−1^ region. The most influential component selected from the loading plot by PCA modeling was predicted by performing PLS analysis, which is one of the unsupervised analysis methods to identify adulteration in ginseng powder. In line with the principle of the PLS, a linear model was constructed for the relationship between the predictive variable group X (spectra data) and the independent variable Y (chemical data). The data were fitted with the PLS method to determine the correlation between infrared spectra in the 3750–550 cm^−1^ region and the values of sample components. Y was computed by Eq. (2):

2 $${\text{Y}} = \beta {\text{X}} + {\text{b}}$$

where β is a vector of regression coefficient, and b is the model offset.

An all-training set model was developed for PLS modeling to predict specific components based on the calibration data and full cross-validation data acquired using 70% of the total samples. The remaining 30% of the total samples was added as input in the training set model to confirm the accuracy of the predicted characteristics of the random sample (Test model). Model accuracy was evaluated using the following parameters: offset, coefficient of determination in calibration (R_c_^2^), coefficient of determination in cross-validation (R_cv_^2^), root mean square error of calibration (RMSEC), and root mean square error of cross-validation (RMSECV) (Giovenzana et al., 2018). R_p_^2^ was used to assess the accuracy of the predictive regression model generated from the remaining data that were not used when developing the calibration model. All multivariate analyses were performed with the chemometric software Unscrambler (version 10.5, CAMO, Trondheim, Norway).

### Statistical analysis

All experimental measurements were taken at least thrice, and the results were presented as mean ± SD. Duncan’s multiple range test (p < 0.05) and Pearson’s correlation analysis were performed using the SPSS software (Statistical Package for Social Sciences, SPSS, Inc., Chicago, IL, USA).

## Results and discussion

### Physicochemical properties

**Fig. 1:**
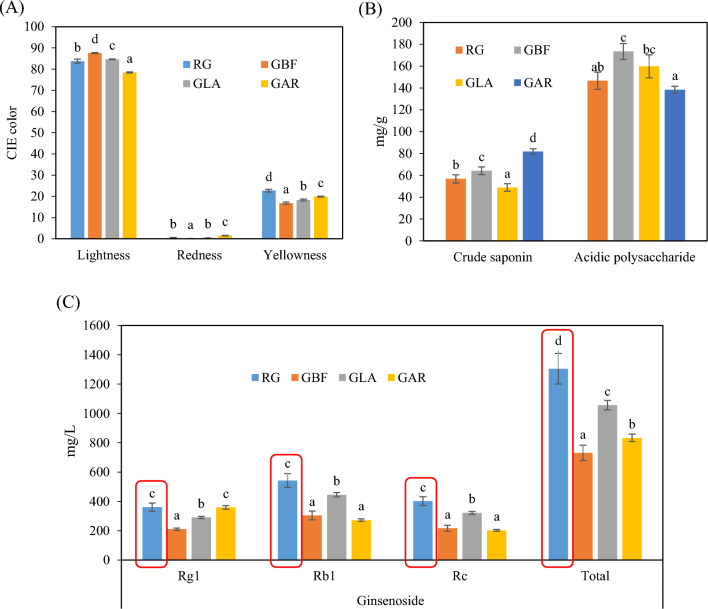
Physicochemical properties of pure ginseng powder and adulterated ginseng powder. (**A**) CIE color; (**B**) Polysaccharides; (**C**) Ginsenosides. *RG* pure ginseng powder, *GBF* ginseng powder adulterated with bellflower root powder, *GLA* ginseng powder adulterated with lance asiabell root powder, *GAR* ginseng powder adulterated with arrowroot powder. Mean ± standard deviation (n = 3), Bars with different superscript letters represent significant differences at 5% significance level

To confirm the presence of adulterants in ginseng powder, it is essential to obtain basic component information of the prepared samples. The CIE L^*^a^*^b^*^ color values and physicochemical properties of various ginseng powders are shown in Fig. 1. RG showed a significantly higher yellowness (22.75) than other samples, but the unique characteristics of RG were not observed in the results of lightness and redness. The color of these powders is determined by the raw material species, harvest time, drying temperature, drying time, and particle size of the powder. Therefore, assessing the adulteration of ginseng powder requires more than simply observing the color and appearance of the powder with the naked eye.

Crude saponin, acidic polysaccharide, and ginsenoside contents of various ginseng powders are shown in Fig. 1B and C. Saponins are composed of a lipid-soluble aglycone consisting of either a sterol or more commonly a triterpenoid and water-soluble sugar residues (Moghimipour and Handali, 2015). Ginseng polysaccharides are generally divided into neutral polysaccharides and acidic polysaccharides. Acidic polysaccharides are polysaccharides with a high content of acidic sugars (Srivastava and Kulshreshtha, 1989). Acidic polysaccharide content analysis indicated that RG, GBF, GLA, and GAR contained 146.55, 173.40, 159.77, and 138.29 mg/g of acidic polysaccharide, respectively. GAR had the highest saponin content at 81.83 mg/g and GLA had the lowest at 48.88 mg/g. The reason for these acidic polysaccharide contents is that heating method, heating time, and heating temperature during processing influence decomposition of sugar, which, in turn, affects the composition of acidic polysaccharide content (Yoon et al., 2005). Additionally, the triterpenoid saponin contained in ginseng also occurs in a wide range of plant species such as lance asiabell, bellflower, soybean, and arrowroot and are distributed throughout the bark, leaves, stems, roots, and even flowers (Kim et al., 2014; Moghimipour and Handali, 2015). Therefore, as the contents of these components are not significantly affected by adulteration, it is inappropriate to use them as indicators of adulteration.

Ginsenoside is a natural steroid glycoside and a type of triterpene saponin. The Rg1, Rb1, and Rc contents of RG were 360.59 mg/L, 542.74 mg/L, and 401.52 mg/L, respectively (Fig. 1C). GBF, GLA, and GAR samples contained 210.22–358.80 mg/L of Rg1, 272.10–445.31 mg/L of Rb1, and 202.39–321.42 mg/L of Rc, respectively. Total ginsenoside content in RG was approximately 19–44% higher than that in the other samples. Ginsenoside can be an important indicator in distinguishing pure ginseng from adulterated samples as it is a unique saponin found in *Panax* plants.

The results of the GC–MS analysis of the volatile flavor components of pure ginseng powder and ginseng powder adulterated with lance asiabell root, arrowroot, and bellflower root are shown in Table 2. Representative sesquiterpene hydrocarbons and sesquiterpene alcohols of ginseng detected in this study included ginsinsene, β-panaginsene, α-neoclovene, and β-famesene (Richter et al., 2005). GC peak areas for ginsinsene, β-panaginsene, and β-elemene of RG were 0.03–0.09%, 0.04–0.22%, and 0.02–0.26%, respectively, higher than those of other samples (GBF and GAR). Additionally, the peak area results for β-gurjunene, α-neoclovene, β-neoclovene, and 1-Dodecene-511-diyne were 0.02–0.16%, 0.05–0.26%, 0.05–0.09%, and 0.18–2.15%, respectively, higher in RG than in other samples. These results demonstrate that RG emits a stronger aroma of ginseng thansamples mixed with other powders, suggesting that aroma component analysis can be useful in distinguishing pure ginseng powder.

**Table 2:** Characteristics of volatile compounds of pure ginseng powder and adulterated ginseng powders

Retention time (min)	Volatile compounds	Area (%)			
		RG	GLA	GAR	GBF
37.34	Ginsinsene	2.22	2.19	2.17	2.13
42.59	β-panasinsene	7.73	7.69	7.65	7.51
46.56	β-Elemene	6.95	6.72	6.93	6.69
46.73	β-Gurjunene	4.86	4.83	4.70	4.72
49.62	α-Neoclovene	8.99	8.94	8.73	8.75
51.03	β-Farnesene	12.50	11.90	12.39	12.52
52.53	β-Neoclovene	1.69	1.64	1.60	1.61
84.31	1-Dodecene-5,11-diyne	2.15	1.86	0.00	1.97

*RG* pure ginseng powder, *GBF* ginseng powder adulterated with bellflower root powder, *GLA* ginseng powder adulterated with lance asiabell root powder, *GAR* ginseng powder adulterated with arrowroot powder

### FT-IR spectra information

**Fig. 2:**
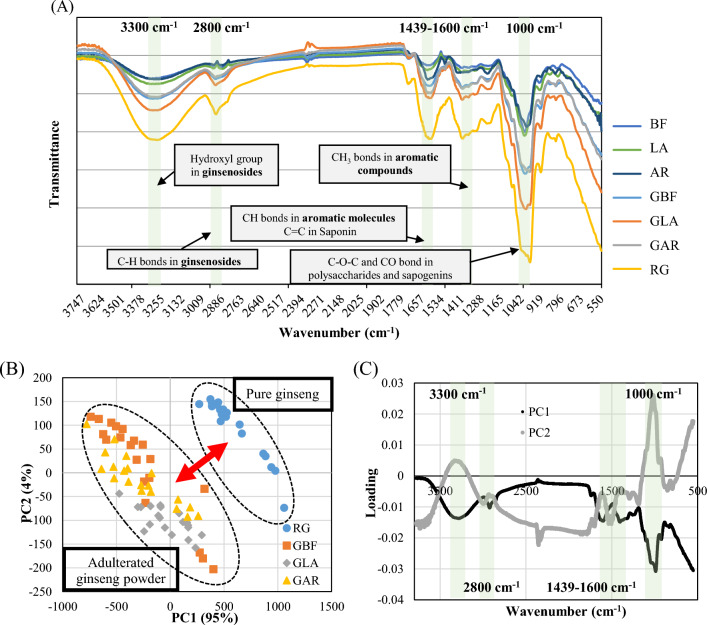
Fourier transform infrared (FT-IR) mean spectra (**A**) from various ginseng powders in the spectral range of 4000–550 cm^−1^, PCA score plot (**B**) as 2D scatter, and loading plot (**C**) of the first two principal components of PCA. *BF* bellflower root powder, *LA* lance asiabell powder, *AR* arrowroot powder, *RG* unadulterated ginseng powder, *GLA* ginseng powder adulterated with lance asiabell root powder, *GBF* ginseng powder adulterated with bellflower root powder, *GAR* ginseng powder adulterated with arrowroot powder

The mean FT-IR spectra from the unadulterated ginseng powder and ginseng powder samples adulterated with BF, AR, LA in the spectral range of 3750–550 cm^−1^ are shown in Fig. 2A. The shape of all spectra looks very similar because most dried root plants are essentially composed of approximately 80% carbohydrate and fiber, ~ 2% of lipid, ~ 5% of nitrogen compounds, and low levels of other components (Choi and Kim, 2011; Hong and Kwon, 2011; Lee et al., 2013; Park et al., 2007;). The graph has prominent valleys in five common regions marked with a pink box. An observation reveals band shifts and changes in the relative bands intensity (absorbance), especially at about 3300, 2900, 1600, 1350 and 1000 cm^−1^. The band at 3309 cm^−1^ represents C–H stretch, the band at 2923 cm^−1^ is due to the stretching vibration of –CH_2_– groups, the 1633 cm^−1^ line is due to the stretching vibration of carbonyl group in the volatile oils and other compounds containing C=O group (Li et al., 2004). The valley at approximately 1350 cm^−1^ has been assigned to the stretching vibration of symmetric COO– groups. Many C–O–C groups exhibit characteristic bands in the 1150–911 cm^−1^ spectral range and generally the strong band at 1026 cm^−1^ is assigned to the vibration of C–O in alcohol hydroxyl group. Differences in relative intensity values were used in the classification model the powder adulterated with BF, AR, and LA, as discussed below.

### PCA score plot and loading plot

Multivariate methods are ideal to provide an effective solution as they allow extracting analytical information from their large spectra regions (Terouzi et al., 2016). PCA score plot presented as a two-dimension scatter diagram simplifies working with multivariate data sets like FT-IR data sets the features extraction as scatter diagram in a two dimensions (Fig. 2B). The four categories of ginseng powders were separated into two clear groups according to the presence of adulteration. The first two PCs were chosen as they explained 99% of the total variability (PC1 = 95%, PC2 = 4%); they made the greatest contribution to detecting adulterated mixtures. PC1 is mainly responsible for the discrimination of all adulterated powder samples from pure ginseng powders. The distances between the distributions of RG and all adulterated samples were similar, and the distributions of GBF, GAR, and GLA slightly overlapped. In conclusion, an exploratory analysis of different ginseng powder samples showed a distinct PCA clustering for samples bearing the label “added other plant,” suggesting the potential use of the method to screen samples for undeclared adulteration.

In this study, loading plot for PCs (Fig. 2C) were chosen for ginseng powder characterization, as PC1 and PC2 preserved complete information regarding the components of ginseng. Wavelengths at pronounced peaks and valleys carried important information in identifying the adulteration and should be selected (Jiang et al., 2020). The four specific transmittance wavenumbers indicated by the pink box were found through an analysis of the value of the loading and the components related to the wavenumbers; 3300 cm^−1^, 3000–2800 cm^−1^, 1600–1400 cm^−1^ and 1000 cm^−1^. The peaks or valleys at 3300 cm^−1^ can be attributed to the stretching of the hydroxyl group in ginsenosides (Kareru et al., 2008), and around 3000–2800 cm^−1^ is related to the presentation of the stretching of C–H bonds in ginsenosides (Kareru et al., 2008). The peak at 1600–1400 cm^−1^ could be assigned to stretching vibration of the carbonyl group (CH bonds in aromatic molecules) in the volatile oils (Li et al., 2004). Ginsenosides, a steroidal saponin conjugated to various sugar moieties and polysaccharides (10–20% by weight), produced the most significant spectral variation in the polysaccharides region (1000 cm^−1^) (Smidt and Meissl, 2007). The characteristic vibrational modes of panaxadiol and panaxatriol saponins (ginsenoside) in ginseng are directly detectable in the crude powder of the medicinal root plant by FT-IR spectroscopy. In summary, results indicate that various types of CH bond and OH bond contained in ginsenoside and aromatic compounds contributed to distinguishing pure ginseng powder from adulterated ginseng, and explicit data are additionally needed to authenticate the authenticity of this supposition.

### Pearson’s correlation

**Fig. 3:**
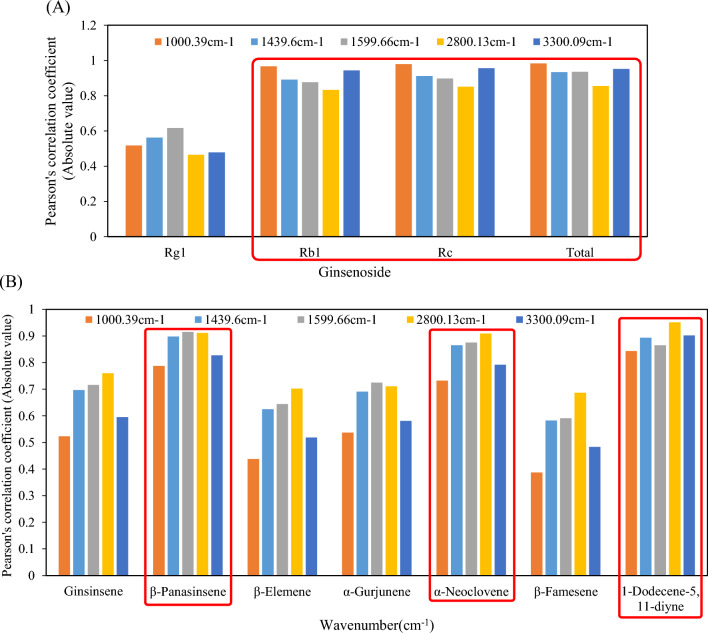
Pearson’s correlation coefficient between the intensity of the mean spectra for key wavenumber and physicochemical data (**A** Ginsenoside; **B** Volatile flavor compounds)

The Pearson’s correlation coefficients between the intensity of the mean spectra for key wavenumber found in the loading plot (Fig. 2B) and physicochemical analysis results are shown in Fig. 3. Correlation analysis was performed because it is important in selecting specific quality indicators for detecting pure ginseng powder. The selected key wavenumbers were: 1000.39, 1439.60, 1599.66, 2800.13, and 3300.09 cm^−1^ based on results in Fig. 2B. Ginsenoside excepted Rg1 showed a high negative correlation of over − 0.95 with spectra at 1000.39 and 3300.09 cm^−1^. Additionally, the correlation coefficient values for β-panasinsene, α-neoclovene, 1-Dodecene-5,11-diyne, and spectral intensity at 2800.13 cm^−1^ were − 0.911, − 0.909, and − 0.951, respectively (results are displayed as absolute values). Therefore, the key wavenumber band selected by the loading plot had a significantly high correlation with ginsenosides (Rb1 and Rc) and flavor components (β-panasinsene, α-neoclovene, and 1-Dodecene-5,11-diyne). These results empirically support the suggestion that these components are the main indicators to distinguish pure ginseng powder.

### Prediction of ginsenosides and volatile flavor compounds in ginseng powder

To improve differentiation of samples, quantitative analysis of ginsenosides (Rb1, Rc, and total ginsenoside) and volatile flavor compounds (β-panasinsene, α-neoclovene, and 1-Dodecene-5,11-diyne) that had the strongest correlation with the FT-IR spectrum was performed using a PLS supervised method (Table 3). The PLS techniques are useful in spectral analysis due to the simultaneous inclusion of many spectral wavelengths instead of the single wavelength used in derivative spectrophotometry (Saad et al., 2010). Among the various pretreatment methods, a great improvement in the precision and predictive abilities of these multivariate calibrations was observed when the derivative function through the Savitzky–Golay algorithm was used. The R_p_^2^ of Rb1, Rc, and the total ginsenoside prediction model (n = 24) for the raw spectra were 0.9452, 0.9419, and 0.8425, respectively. The R_p_^2^ of Rb1, Rc, and the total ginsenoside prediction model for the SG first derivative preprocessing spectrum were 0.9650, 0.9635, and 0.9129, respectively, which were higher than those of the prediction results for raw data. In contrast, the R_p_^2^ of SNV and MSC preprocessed data for ginsenoside content was 0.8278–0.8930, which was lower than that of raw data (0.9129–0.9650), suggesting that it was inappropriate to apply to the prediction model of ginsenoside content in various ginseng powders. Additionally, the R_p_^2^ of the volatile flavor prediction model developed with the Savitzky–Golay first-order differential preprocessing spectrum was high (approximately 0.94 or more). Inagaki et al. (2019) and Xu et al. (2014) also estimated the ginsenoside content of ginseng through spectroscopic data. The findings of this study confirm that it is possible to evaluate the contents of ginsenoside and volatile flavor compounds of ginseng powder adulterated by addition of other plants, and unlike previous studies, the model developed in this study can bea basis for exploring pure ginseng powder. Further studies involving a larger number of ginseng products and ginseng samples adulterated with other plants could improve the accuracy of the model developed in this study.

**Table 3:** Accuracy of the PLS model for ginsenoside and volatile flavor compounds prediction according to pretreatment methods

		Pretreatment	Factor	Training model				Prediction model
				Calibration		Cross-validation		
				RMSEC	R_c_^2^	RMSECV	R_cv_^2^	R_p_^2^
Ginsenoside	Rb1	Raw	7	17.1083	0.9755	34.5285	0.9036	0.9452
		Smoothing	7	17.1175	0.9754	34.5343	0.9035	0.9451
		SNV	4	28.7936	0.9305	35.1821	0.8999	0.8278
		MSC	7	18.3743	0.9717	26.6486	0.9426	0.8875
		SG-1	5	16.0111	0.9785	24.3938	0.9519	0.9650
	Rc	Raw	7	12.4639	0.9765	25.3587	0.9061	0.9419
		Smoothing	7	12.4735	0.9765	25.3693	0.9060	0.9419
		SNV	4	21.5124	0.9300	26.2647	0.8993	0.8335
		MSC	7	13.7182	0.9715	19.8909	0.9422	0.8930
		SG-1	5	11.1554	0.9812	17.4240	0.9557	0.9635
	Total	Raw	4	41.1480	0.9653	48.3807	0.9537	0.8425
		Smoothing	4	41.1558	0.9653	48.3868	0.9537	0.8425
		SNV	4	54.6226	0.9388	64.1535	0.9186	0.8554
		MSC	5	51.5974	0.9454	60.0434	0.9287	0.8411
		SG-1	5	28.0526	0.9839	43.7718	0.9621	0.9129
Volatile flavor compounds	Panasinsene	Raw	7	0.0184	0.9510	0.0236	0.9215	0.8709
		Smoothing	7	0.0184	0.9509	0.0237	0.9214	0.8709
		SNV	7	0.0174	0.9559	0.0255	0.9087	0.8653
		MSC	7	0.0155	0.9653	0.0235	0.9228	0.9032
		SG-1	6	0.0123	0.9781	0.0241	0.9185	0.9591
	Neoclovene	Raw	7	0.9376	0.9319	1.2247	0.8880	0.9278
		Smoothing	7	0.9386	0.9318	1.2254	0.8878	0.9278
		SNV	7	0.9119	0.9356	1.4575	0.8413	0.8601
		MSC	7	0.8748	0.9407	1.4739	0.8377	0.8517
		SG-1	5	0.6581	0.9665	1.1460	0.9019	0.9563
	1-Dodecene-5,11-diyne	Raw	7	0.1341	0.9762	0.1747	0.9610	0.9212
		Smoothing	7	0.1342	0.9762	0.1748	0.9610	0.9211
		SNV	5	0.1654	0.9638	0.2262	0.9347	0.9560
		MSC	5	0.1786	0.9578	0.2618	0.91252	0.9607
		SG-1	4	0.1555	0.9680	0.2197	0.9384	0.9432

*SNV* standard normal variate, *MSC* multiplicative scatter correction, *SG-1* Savitzky–Golay first derivative

## Conclusion

This study proposed the application of FT-IR and multivariate analysis for the detection of adulteration in ginseng powder. Pure ginseng powder (RG) and three ginseng powders adulterated with bellflower root, lance asiabell root, and arrowroot were distinguished and identified using PCA, and PLS analysis. The PCA model realized the initial division between pure ginseng powder and adulterated ginseng powders. Based on the loading plot results, ginsenosides (Rb1 and Rc) and volatile flavor compounds (β-panasinsene, α-neoclovene, and 1-Dodecene-5,11-diyne) were the most influential discriminating parameters in separately clustering RG and adulterated RG. The PLSR models for predicting ginsenoside and flavor compounds contained in RG and adulterated RG were fitted based on the optimal pretreatment methods (Savitzky–Golay first derivative) with R^2^ values of 0.9129–0.9650 and 0.9432–0.9591, respectively. These results showed that the suggested tool is dependable and can be used to verify the authenticity of ginseng powder and predict the degree of adulteration of adulterated samples, and can be applicable to check the authenticity or degree of purity of other food or agricultural products.
